# A Supplementary Description of *Cypridina mariae* and Rediagnosis of the Genus *Cylindroleberis* (Ostracoda: Myodocopa: Cylindroleberididae)

**DOI:** 10.1371/journal.pone.0001960

**Published:** 2008-04-16

**Authors:** Anna E. Syme, Gary C. B. Poore

**Affiliations:** Museum Victoria, Melbourne, Victoria, Australia; Paleontological Institute, Russian Federation

## Abstract

The ostracod family Cylindroleberididae is based on the genus *Cylindroleberis* Brady, 1868, and has a complicated nomenclatural history. The type species of *Cylindroleberis* is *Cypridina mariae* Baird, 1850. Baird described only the carapace, which had been considered lost. Thus, there was no reference point for the concept *C. mariae* or the genus *Cylindroleberis.* Baird's material has now been found in the Natural History Museum, London, U.K., and is illustrated here. To clarify the taxonomic status of *C. mariae* and *Cylindroleberis*, specimens were obtained from near the type locality, and a supplementary description is presented. This includes description of appendages, particularly the first antenna and mandible, which contain important diagnostic characters. This supplementary description provides important information about *C. mariae*, allowing a revision of the genus *Cylindroleberis*, and establishing a framework for future biological research on this ostracod group.

## Introduction

Cylindroleberidid ostracods are distinguished from other myodocopid families by their flat gills at the posterior of the body [1]–[3]. A cladistic analysis based on morphological and molecular characters suggests cylindroleberidids are a monophyletic clade within the Myodocopa [4]. The Cylindroleberididae have a global marine distribution and range from the intertidal to depths of 4500 metres [2]. The complicated taxonomic history and current species list of Cylindroleberididae was presented in Syme and Poore [5].

The Cylindroleberididae and Cylindroleberidinae are based on the genus *Cylindroleberis* Brady, 1868 [6]. The type species of *Cylindroleberis* is *Cypridina mariae* Baird, 1850 [7], subsequently designated by Sylvester-Bradley [8] despite the inadequacy of Baird's description. Like many ostracod descriptions in the nineteenth century, Baird's description was of the carapace only. Although Baird's specimens were considered lost [9], the material is stored in the Natural History Museum, London, U.K. We have examined this material and discuss it below.

**Figure 1:**
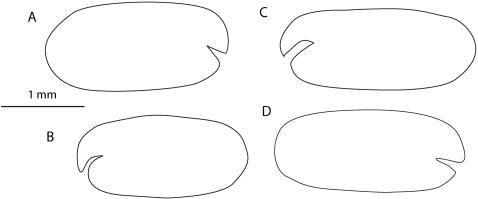
*Cylindroleberis mariae,* specimen number BMNH:1945.9.26.101-104. A, carapace of holotype; B-D, carapaces of additional material from Shetland Islands.

Whilst other specimens have been described under the name *C. mariae*, we reject their synonymy for the following reasons. Brady [10] illustrated and briefly described the carapace and limbs of an ostracod that he called *C. mariae*. Skogsberg [9] considered the figures of the limbs to be “incomplete and incorrect”, although he did not elaborate on the particular errors. The general problems of the figures are the apparently incorrectly-placed setae ( =  bristles) (his Fig 1a), lack of some sutures (Fig 1b), truncation (Fig 1i), and uncertainty about whether the mandible, maxilla (fourth limb), fifth, sixth, seventh limbs and furca are from the male or female specimen (not stated in figure captions). More specific problems are the inability to determine the status of generic diagnostic characters: whether the s-seta of the first antenna has a proximal filament and whether the mandible has a lateral e-seta. Because of this, we agree with Skogsberg's view that Brady's specimens do not permit a certain identification and are not clearly referable to any other known material.

Brady and Norman [11] also illustrated specimens identified as *C. mariae.* Their figures are of an adult male and a juvenile male. The latter is referred to as a female, but the endopod of the second antenna has the typical “robust” form of juvenile males in the family Cylindroleberididae. Because of uncertainty in determining the conspecificity of the sexes, we do not consider these specimens as synonymous with Baird's concept of *C. mariae*. Other references to *C. mariae* by Cushman [12] and Juday [13] are of American species and so are not near the type locality for *C. mariae* Baird. Muller's [14] specimen of *C. mariae* was synonymized into of *C. grimaldi vicina* by Skogsberg [9].

*Cylindroleberis mariae* appears in various electronic databases and recent literature[15]. We consider a supplementary description necessary to clarify the nominal taxon *Cypridina mariae* (now *Cylindroleberis mariae*) and because it has an impact on the accepted definition of *Cylindroleberis*.

The type locality of *Cypridina mariae* Baird is “off the Isle of Skye”, Scotland, U.K. Of the available material in the National Museum of Scotland, Edinburgh, specimens from the Shetland Islands, were chosen for examination. These are described herein. Besides Baird's material, the only illustrated specimens are carapaces from a male and female from Norway [8], which are no more informative. The supplementary description herein is of an adult female and juvenile male consistent with what little is known from Baird's material and description of the holotype (carapace shape). The description fits with the current diagnosis of *Cylindroleberis* except for the arrangement of filaments on the s-seta, and thus the genus diagnosis of *Cylindroleberis* is expanded to include the variation in this character.

The aim of this project was to clarify the morphology of the species *C. mariae* and, as a consequence, the genus *Cylindroleberis*. The illustration of the type material of *C. mariae*, along with a detailed supplementary description, clarifies important morphological characters of that species, and the genus *Cylindroleberis* has been revised in that context.

## Results

### Systematics

***Cylindroleberis*** **Brady, 1868**

*Asterope* Philippi, 1840: 186. [16]

*Cylindroleberis* Brady, 1868: 127 [6]

*Asteropina* Strand, 1928 [17] [unnecessary replacement name, *Asterope* preoccupied]

*Polyleberis* Kornicker, 1974: 48 [18] [new synonym]

Type species: *Cypridina mariae* Baird, 1850 [7], subsequent designation by Sylvester-Bradley [8].

Emended diagnosis (adult female): Carapace elongate (height <50% of length), first antenna s-seta with proximal+distal filament configuration as 1+6 or 0+7 to 0+9, first antenna d-seta absent, mandible exopod less than 25% length of first endopod article, mandible e-seta absent.

Remarks: The genus was rediagnosed in detail by Skogsberg [9] (as *Asterope*) and Poulsen [19] (as *Asteropina*). Poulsen's diagnosis defined the adult female s-seta as having a filament configuration of 1+6, which is true in all known species. *Cylindroleberis mariae* has a configuration of 0+9, and the A-1 male with 1+6. Historically, the presence or absence of a proximal filament on the s-seta has been considered a good generic character. However, within the family, the proximal filament varies continuously from absent to short to long, and the pattern has been interpreted differently. A “long” proximal filament can alternatively be interpreted as a “short” terminal filament. For example, species in the genus *Bathyleberis* Kornicker, 1975 [1], all with 7 filaments in total, show the full range of this character, with some described as 1+6 and some as 0+7. Further, the ontogeny of this character is not clear: juvenile females may lack the proximal filament where adults have it (*Cylindroleberis vibex* A-2 instar), and the reverse (*Synasterope calix* A-2 instar) [20]. The A-1 male of *C. mariae* described below with 1+6 differs in this character from the female.

Thus, the generic diagnosis of *Cylindroleberis* is expanded to include s-seta arrangements of either 1+6 or 0+7–9 filaments, i.e., between 7–9 filaments in total. The generic diagnosis of *Polyleberis* (monotypic: *Polyleberis mackenziei* Kornicker, 1974 [18]) includes an s-seta with 0+7 to 0+9 filaments, (differing between individuals in the species). Because this definition includes *C. mariae*, we consider it no longer justified to keep *Polyleberis* separated from *Cylindroleberis* on the basis of this character alone, and it is synonymized herein.

Included species: The composition of *Cylindroleberis* was discussed by Kornicker [21]. There are currently 14 species in the genus: *C. bacescui* Kornicker and Caraion, 1974 [22]; *C. grimaldi* (Skogsberg, 1920) [9]; *C. kliei* Kornicker, 1976 [23]; *C. mariae* (Baird, 1850) [7]; *C. marranyin* Syme and Poore, 2006 [24]; *C. minuta* (Poulsen, 1965) [19]; *C. nodulifera* (Poulsen, 1965) [19]; *C. thailandica* (Poulsen, 1965) [19]; *C. variabilis* Kornicker, 1970 [25]; *C. verrucosa* (Poulsen, 1965) [19]; *C. vibex* Kornicker, 1992 [20]; *C. vix* Kornicker, 1992 [20]; *C. mackenziei* (Kornicker, 1974)[18] and *C. vicina* (Skogsberg, 1920) [9].

*C. vicina* was previously a subspecies *C. grimaldi vicina* (Skogsberg, 1920) [9]. However, we believe its differences from *C. grimaldi* warrant species status, and here raise it to the species rank as *C. vicina*. It is diagnosed by no setae on mandiblular basale dorsal margin at midlength, a carapace length greater than 1.5 mm, and fewer than 5 anteroventral setae on the sixth limb. *C. nodulifera* and some specimens of *C. variabilis* also lack setae at this position on the mandibular basale; these species are smaller than *C. vicina* (carapace length less than 1.5 mm). *C. marranyin* also lacks setae at this position on the mandibular basale, but has greater than 5 anteroventral setae on the sixth limb.

A further species, C. *rangiroaensis* Hartmann, 1984 [26], has a long mandibular exopod which excludes it from *Cylindroleberis* [21] . Here, we place it in *Synasterope* Kornicker, 1975 [1], because it shares with other members of this genus: first antenna s-seta with proximal+distal filament configuration 0+6, first antenna d-seta absent, mandible exopod greater than 50% length of first endopod article, and mandible e-seta absent.

***Cylindroleberis mariae*** **(Baird, 1850)**

*Cypridina mariae* Baird, 1850: 257, plate XVII, figs. 5–7 [7].

*Cylindroleberis mariae*.— Sylvester–Bradley, 1961:Q402, figs. 3a–d [8].

*Asteropina mariae*.—Strand, 1928: 30 [17].

not: *Asterope mariae*.—Brady, 1871: 289 [27].

not: *Cylindroleberis mariae*.—Brady, 1868 [10]; Brady and Norman, 1896 [11]; Cushman, 1906 [12]; Juday, 1907 [28]; Muller, 1912 [14].

Material examined. – BMNH (Natural History Museum, London, United Kingdom).

Holotype: BMNH:1945.9.26.101-104, one dried carapace in a pill box, length 2.17 mm, height 0.91 mm (Figure 1). Written on the back of the pillbox is: “Cypridina mariae Baird, off the Isle of Skye, R.M. Andrew Esq [???] 1850”. Question marks denote uncertain text.

Additional material: with same number, BMNH:1945.9.26.101-104, three dried carapaces in one pill box (Figure 1). Written on the back of the pillbox is: “Cypridina mariae Baird, St Magnus' Bay, Shetlands 1867, J.G. Jeffrey, Esq.”

Material examined. – NMSZ (National Museum of Scotland, Edinburgh).

Specimens for supplementary description: NMSZ:1996.004.293 adult female on slide, Shetland Islands, west coast, UK, 60°22.5′N, 01°32.48′W, 132.2 m depth, collected on benthos surface using a Day grab, Braer survey. NMV (Museum Victoria, Australia) J53222 A-1 female on slide; NMSZ:1996.004.427 A-1 male on slide; from same station locality.

Other material: NMSZ:1996.004.428 3 undissected specimens in 70% alcohol; NMV J53223 1 undissected specimen in 70% alcohol, from same station locality.

Diagnosis.– first antenna s-seta with 0+9 filaments, furca with 10 claws/setae.

Supplementary description of adult female.— NMSZ:1996.004.293 except where noted otherwise.

**Figure 2:**
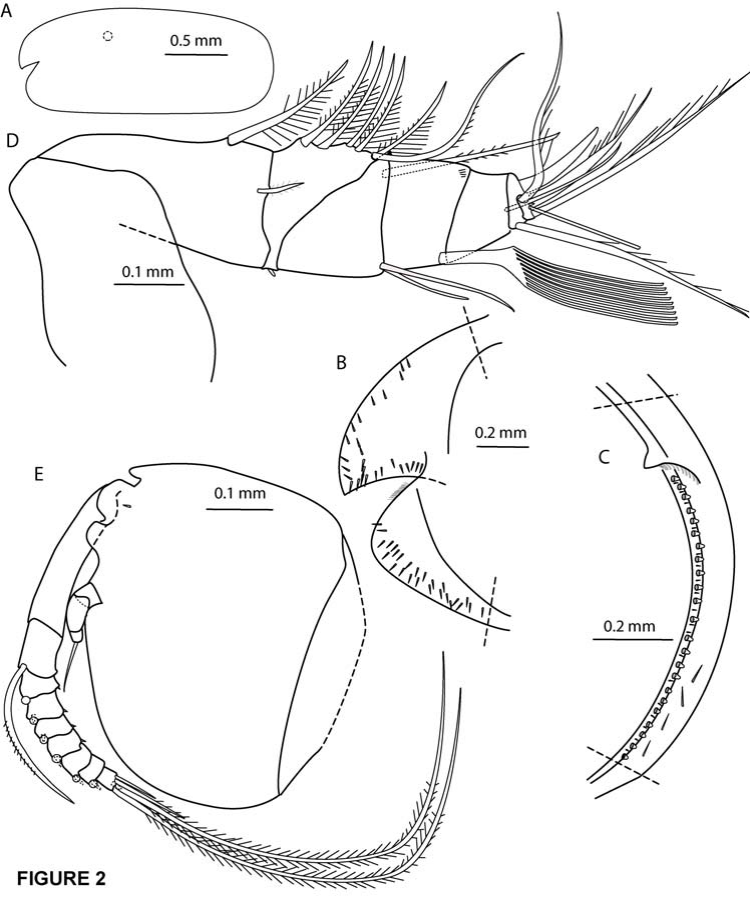
*Cylindroleberis mariae*, adult female, specimen number NMSZ 1996.004.293. A, carapace outline, left, l.v. (lateral view), position of lateral eye dotted; B, inner right valve, anterior; C, inner right valve, posterior; D, first antenna, right, l.v.; E, second antenna, right, m.v. (medial view), bases of setae on exopod articles 3–8 represented by circles.

Carapace: elongate, incisur at midheight, posterior end evenly rounded (Figure 2A), length 2.15 mm, height 0.89 mm. Selvage: Fringe of hairs at inner end of ventral margin of incisur. Infold: anterodorsal (rostral) infold with 26 setae; anteroventral infold with 26 setae between list and valve edge (Figure 2B), narrow list continuing ventrally, list broadening slightly at posterior infold with 24 broad triangular transparent setae and 18 smaller setae placed between, and 4 setae between list and valve edge (Figure 2C).

First antenna (Figure 2D): Article 2 with 1 spinous dorsal seta and 1 lateral seta with faint spines. Article 3 with 1 short ventral seta and 6 dorsal setae–all setae with long spines except seta 3 (no spines) and seta 6 (shorter spines). Article 4 with 1 dorsal medial seta with short marginal spines and 2 ventral setae. Article 5 with sensory seta with no short proximal and 9 terminal filaments. Article 6 with medial seta with faint spines, reaching tip of a-claw. Article 7 with a-claw, b-seta with 5 marginal filaments, c-seta with 6 marginal filaments. Article 8 with minute peg d-seta, e-seta bare with blunt tip, f-seta bent dorsally with 4 marginal filaments, g-seta with 6 marginal filaments.

Second antenna (Figure 2E): Protopodite with small bare distal medial seta. Endopodite with 3 articles, end article with terminal filament. Exopodite: article 2 with seta with marginal spines, reaching 8th article. Articles 3–8 with long setae with marginal spines, articles 4–8 with basal spines. Article 9 with larger basal spine, 2 shorter setae and 2 long setae with marginal spines.

**Figure 3:**
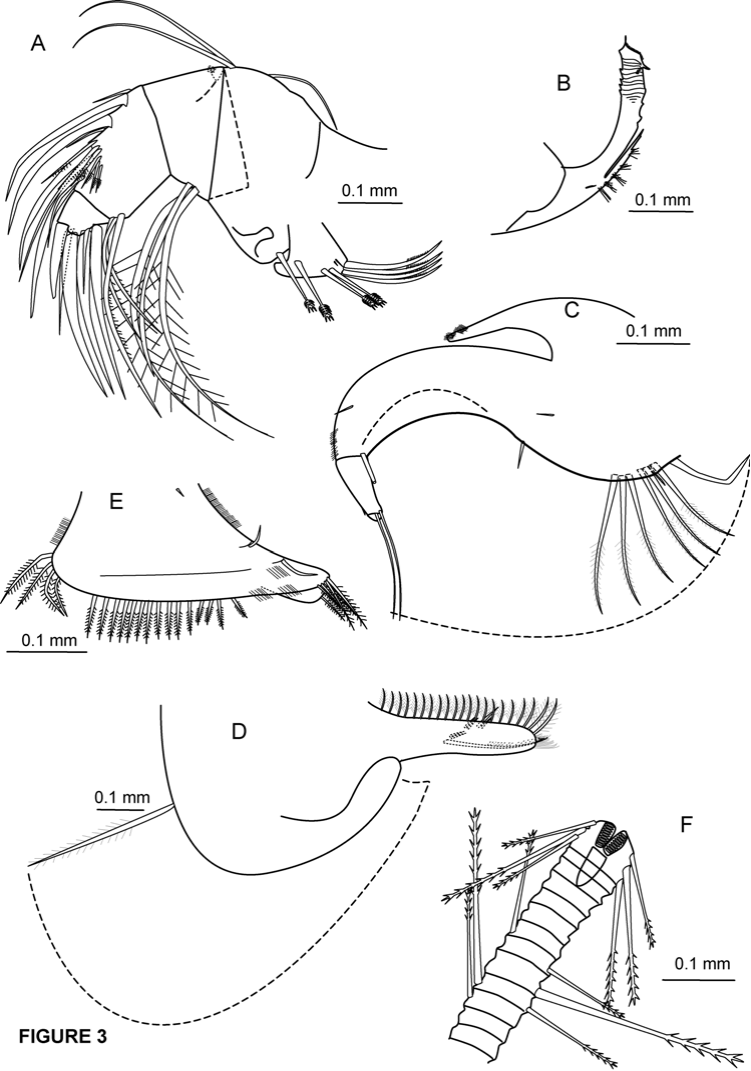
*Cylindroleberis mariae*, adult female, specimen number NMSZ 1996.004.293. A, mandible, right, m.v. *Cylindroleberis mariae*, adult female, NMV J53222; B, coxale endite of mandible, right, m.v. *Cylindroleberis mariae*, adult female, specimen number NMSZ 1996.004.293; C, maxilla (fourth limb), left, l.v., setal comb setal limits shown by dotted lines, basale ventral distal seta broken; D, fifth limb, right, l.v., setal fan limits shown by dotted lines; E, sixth limb, left, m.v.; F, seventh limb.

Mandible (Figure 3A): coxale endite: broken, for specimen NMV J53223, coxale endite with small seta near base of ventral branch; ventral branch with clustered spines; dorsal branch with distal serrations and terminal tip (Figure 3B). Mandible basale endite: with 4 spinous end setae, 3 triaenid setae with 3–4 paired spines excluding terminal pair, 2 dwarf setae of unequal length. Mandible basale: ventral margin with 1 triaenid seta with 2 pairs of spines excluding terminal pair, proximal to U-shaped boss; dorsal margin with 1 seta just distal to midlength and two long bare terminal setae, equilength. Mandible exopod with hirsute tip, exopod length 10% of dorsal margin of first endopod article. Mandible endopod: 1st endopod article with 3 long ventral setae (1 with short spines, 2 with long spines). 2nd endopod article: ventral margin with 3 long terminal setae with short spines, dorsal margin with stout a-, b-, c-, and d-setae, 1 slender seta proximal to a-seta; medial side with 7 “cleaning” setae, and 1 long g-seta distal to base of d-seta; lateral side with no e-seta between b- and c- setae, and 1 long f-seta between c- and d- setae. 3rd endopod article with stout dorsal claw, 3 stout setae with faint spines, and 2 slender shorter setae, the longer one attached laterally.

Maxilla (fourth limb) (Figure 3C): with setal comb attached to lateral ventral side of maxilla basale. Triangular epipod. Endite I (proximal) with 3 long spinous setae and 1 short bare seta. Endite II (distal) with 3 long spinous setae. Basale with 1 proximal lateral seta, ventral margin with 1 proximal seta and 1 long distal seta with faint spines, dorsal margin with 1 distal seta. 1st endopod article with small alpha seta, 1 long beta-seta. 2nd endopod article with long terminal seta.

Fifth limb (Figure 3D): ventral section with fan of long setae. Side “comb” with long exopod seta, three pairs of shorter lateral setae near ventral margin of comb, dorsal margin of comb with hairs at distal end. Note: in the “life” position, the comb is folded; on the slide preparation the comb has flipped back; however, the orientation terms “dorsal” and “ventral” apply to the position of the comb when in the life position.

Sixth limb (Figure 3E): Small seta on proximal medial side; anterior margin with seta at each endite suture, lateral flap of skirt with 6 setae, ventral margin with 15 setae with spines, posteroventral corner with 4 plumose setae. Seventh limb (Figure 3F): with 12 setae, each with 4–7 bells. Combs forming acute angle, each comb with 9 teeth.

**Figure 4:**
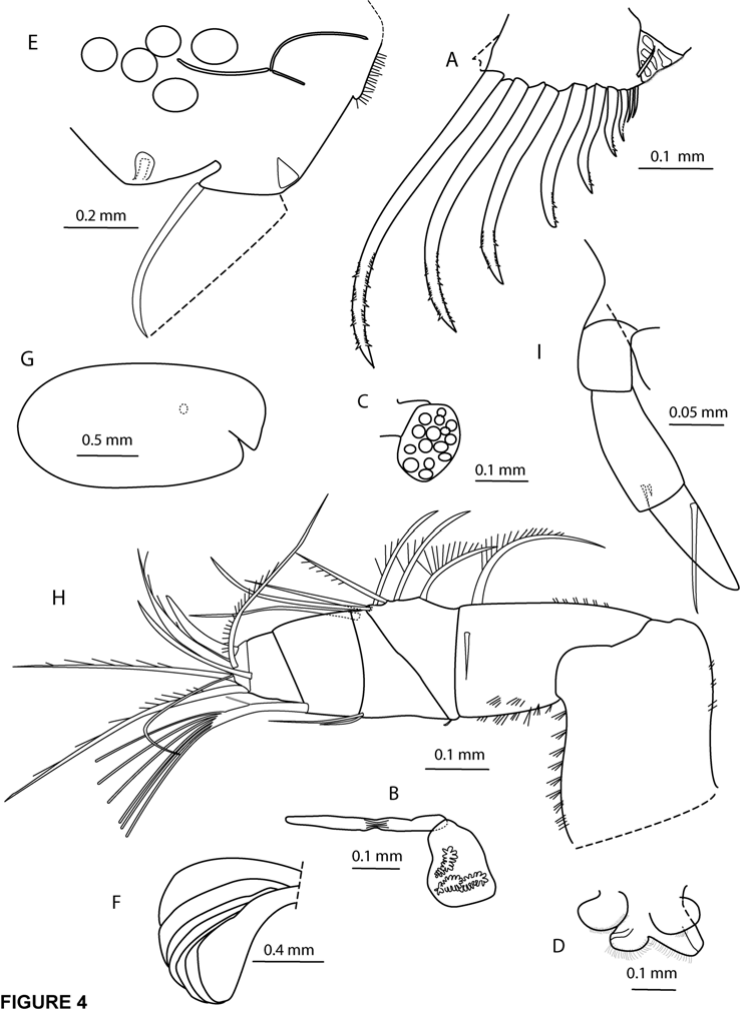
*Cylindroleberis mariae*, adult female, specimen number NMSZ 1996.004.293. A, furca, left lamella, l.v.; B, medial eye and Bellonci organ; C, lateral eye; D, upper and lower lips, dorsal view; E, posterior of body, left, l.v., reproductive organ dotted, eggs (or parasites) shown as circles; F, gills, right lamella, l.v. *Cylindroleberis mariae*, A-1 male NMSZ:1996.004.427; G, carapace outline, right, l.v., position of lateral eye dotted; H, first antenna, left, l.v.; I, second antenna endopod, left, m.v.

Furca (Figure 4A): Each lamella with 9 claws/setae decreasing evenly in size, and 1 lateral seta pointing posteriorly. Bellonci organ (Figure 4B): narrowed in middle. Medial eye (Figure 4B): unpigmented, bare. Lateral eye (Figure 4C): with 15 ommatidia. Lips (Figure 4D): upper lips hirsute lobes, no anterior spines visible on dorsal margins. Lower lips hirsute flaps. Posterior of body (Figure 4E): Hirsute with slightly-rounded lower section of posterior body; no thumb-like dorsal process; 5 embryos (or possibly parasites) observed on lateral side of body; reproductive organs paired oval pores anterior to furca. Gills (Figure 4F): 7 pairs of flat gills at posterior of body.

Supplementary description of A-1 Male.— NMSZ:1996.004.427. The specimen is considered to be an A-1 male due to the robust (not recurved) form of the second antenna endopod. Notable differences from the adult female are presented here:

Carapace (Figure 4G): length 1.93 mm, height 0.96 mm. First antenna (Figure 4H): article 5 sensory seta with 1 short proximal and 6 terminal filaments. Second antenna endopod (Figure 4I): article 2 with 2 short lateral setae, article 3 with 1 long medial seta. Mandible exopod length: 50% of dorsal margin of first endopod article. Maxilla (fourth limb): Basale dorsal margin with 1 seta at midlength and 3 distal setae. Furca: with 8 claws/setae decreasing evenly in size, no lateral seta pointing posteriorly. Lateral eye with 19 ommatidia.

Distribution. – Known from Isle of Skye (unknown depth) and Shetland Islands (132.2 m depth), Scotland, U.K.

## Discussion

The supplementary description herein agrees with the size and shape of the holotype, and with Baird's description [7], which states “carapace valves elongate, oval, of exactly the same size at each extremity; extremities rounded. Dorsal and ventral margins nearly plane, or very slightly arched”, “notch or ventral margin of anterior extremity blunt, leaving the upper and lower margins of the notch very obtuse”.

*Cylindroleberis mariae* is similar to *Polyleberis mackenziei* Kornicker, 1974 [18]. The holotype of *P. mackenziei* was described [18] but was considered not to be mature. The type locality is Gulf of Naples, Italy. Further specimens were described later from Mauritania and descriptions provided of an adult female and juvenile male [22]. The carapace of *C. mariae* is slightly longer than that of *P. mackenziei*–2.15 mm compared to the longest *P. mackenziei* specimen recorded of 1.96 mm. There are 10 furcal claws compared to 9, and there is no anterior spine on the upper lip. The A-1 male of *C. mariae* described here has an s-seta configuration of 1+6. This is similar to the juvenile male of *P. mackenziei* which is described as having a configuration of 0+7, with the first terminal filament being half the length of the others. The differences are insufficient to warrant a generic distinction.

Apart from the different s-seta configuration, *Cylindroleberis mariae* can be distinguished from other species of *Cylindroleberis* in carapace size (only *C. vix* also has a carapace length of greater than 2 mm in the adult female), and the presence of the distomedial seta on the second antenna protopod (only present in *C. vibex*).

This supplementary description allows clarification of the attributes of *C. mariae*, and reduces uncertainty in the concept of this species. With this information, the genus *Cylindroleberis* is also revised, providing a sound taxonomic framework for ecological, physiological and evolutionary research on this ostracod family.

## Materials and Methods

Material was examined under compound and dissecting microscopes (up to X600 magnification). Dissections were made using tungsten needles, and appendages were mounted on microscope slides. Pencil illustrations were made using a camera lucida, and were then scanned and digitally traced using Adobe Illustrator software.
